# Investigation into the anti‐inflammatory properties of metformin in intervertebral disc cells

**DOI:** 10.1002/jsp2.1197

**Published:** 2022-03-10

**Authors:** Rahul Ramanathan, Ayesha Firdous, Qing Dong, Dong Wang, Joon Lee, Nam Vo, Gwendolyn Sowa

**Affiliations:** ^1^ Ferguson Spine Laboratory, Department of Orthopaedic Surgery University of Pittsburgh Pittsburgh Pennsylvania USA; ^2^ Department of Physical Medicine and Rehabilitation University of Pittsburgh Pittsburgh Pennsylvania USA

**Keywords:** AMPK pathway, anti‐inflammatory, anti‐inflammatory therapy, biomechanical profile of intervertebral disc, disc aging, disc mechanics, inflammatory profile of intervertebral disc, intervertebral disc degeneration, metformin therapy, NF‐kB translocation

## Abstract

**Introduction:**

Intervertebral disc degeneration (IDD) is closely related to heightened inflammation in the annulus fibrosis (AF) and nucleus pulposus (NP) cells in the intervertebral disc. An imbalanced matrix homeostasis has been shown to contribute to disc degeneration and associated discogenic low back pain. Metformin, a diabetes medication, has been noted to exhibit anti‐inflammatory properties through upregulation of the AMPK pathway, leading to various anti‐inflammatory‐related responses in hepatocytes. However, it is still unclear how metformin influences disc cellular response to inflammatory stress and the corresponding mechanism. Hence, the objective of this study is to elucidate the effects of metformin on expression of key pro‐inflammatory, catabolic, and anabolic factors within rat AF cells in response to inflammatory stimulation and mechanical tensile stress.

**Methods:**

Five Fischer 344 rats were sacrificed and their spines isolated. AF cells were cultured and plated in flexible silicone membrane‐based six‐well plates. Wells were split into eight groups and subjected to metformin, IL‐1β, mechanical stretch, and combined treatments. Relative gene expressions of MMP‐13, COX‐2, iNOS, AGC, and Col1 were assessed with quantitative real‐time polymerase chain reaction (qRT‐PCR), and downstream prostaglandin E_2_ (PGE2) production was quantified with enzyme‐linked immunosorbent assay (ELISA). NF‐kB nuclear translocation was also quantified.

**Results:**

Metformin in the presence of the combined stress treatments (M + IL/S) significantly increased Col1, COX‐2, and MMP‐13 gene expression, decreased PGE2 production compared to IL/S conditions alone. Metformin treatment of cultured rat annulus fibrosus cells significantly reduced the nuclear translocation of NF‐κB after 4 h of IL‐1β treatment from 43.1% in case of IL‐1β treatment down to 26.2% in the case of metformin + IL‐1β treatment.

**Discussion:**

The lack of metformin‐mediated suppression of inflammatory response in the nonstretch groups indicates that metformin may be enacting its effects through a stretch‐dependent manner. These results suggest a foundation for pursuing further research into metformin's potential role as an anti‐inflammatory agent for curtailing intervertebral disc degeneration.

## Graphical abstract

Metformin enacts its anti‐inflammatory effects on intervertebral disc cells in a stretch‐dependent manner, revealing its potential as a therapeutic agent for back pain

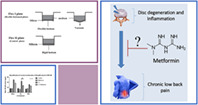

## 1 INTRODUCTION

Lower (lumbar) back pain is one of the most common symptoms leading to a clinical visit. In 2012, the estimated annual direct medical cost for all back‐related conditions was $253 billion.^1^ Particularly in the geriatric population, intervertebral disc degeneration (IDD) has been documented to play a contributive role in the etiology of chronic lower back pain.^2^ Multiple lines of evidence exist for the role of inflammation in IDD. Tisherman et al. noted that inhibiting NF‐κB, a central mediator of cellular response to inflammation,^3^ dampens the inflammatory response and catabolic factor production in the intervertebral disc cells,^4^ implicating a possible role for this pathway in IDD.^5^

Downstream of NF‐κB signaling, the inflammatory mediator, prostaglandin E_2_ (PGE_2_) is of particular interest in the study of IDD. Previous groups have shown that mechanical tensile strain and inflammatory factors, such as IL‐1β and TNF‐α synergistically increase PGE_2_ concentration many‐fold within AF cells.^6^ PGE_2_ is reported to perturb matrix homeostasis in human nucleus pulposus cell culture.^7^ There is overwhelming evidence to suggest that depletion of PGE_2_ or suppression of COX‐2, the enzyme that synthesizes PGE_2_, reduces the inflammatory response in disc cells.

This inflammatory pathway is also mediated by mechanical stretch. Miyamoto's group has demonstrated mechanical stretch‐mediated upregulation of PGE_2_ and COX‐2 expression.^6^ Tensile stress also leads to increased expression of proteases activated under conditions of inflammation, such as MMPs which have been implicated in degeneration of discs and noted in elderly patients with advanced disc degeneration.^8^ Mechanical stretch, in conjunction with IL‐1β treatment, has been found to enhance PGE_2_ synthesis beyond either treatment alone in AF cells.^6^ This combination of stimuli exacerbates the local inflammatory response potentially accelerating the rate of IDD.

As inflammation plays a large role in IDD, anti‐inflammatory agents are currently gaining ground in becoming therapeutic precursors for treating IDD‐induced low‐back pain. Metformin is a widely used oral medication for type II diabetes that has been shown to have anti‐inflammatory effects in multiple tissues. Although the exact mechanism in which metformin acts is still largely unknown, Zhou et al. demonstrated that metformin is correlated with increased activation of the AMP‐activated protein kinase (AMPK) pathway in primary hepatocytes.^9^ While the AMPK pathway is largely a homeostatic pathway upregulating glucose and fatty acid uptake when cellular energy is low, it has also been shown to modulate multiple different aspects of cellular metabolism including autophagy, redox regulation, aging, and inflammatory response.^10^ In the retinal vascular endothelial cells, metformin has been shown to downregulate NF‐κB and inflammatory markers.^11^ In bovine retinal capillary and endothelial cells, metformin acts through the suppression of ROS signaling.^12^ Metformin has also been shown to inhibit NF‐κB in rabbit endothelial cells,^13^ human umbilical vein endothelial cells,^13^ and human vascular smooth muscle cells.^14^ Metformin also inhibits NF‐κB in macrophages^15^ and in human adipocytes,^16^ and suppresses ROS activation in mouse lung cells.^17^, ^18^

In the context of IDD, metformin has been found to increase AMPK activation, which has multiple downstream benefits. Namely, AMPK inhibits NF‐κB translocation into the nucleus via SIRT1/FOXO/PGC1α, downregulating the transcription of pro‐inflammatory genes, such as COX‐2 and iNOS. Metformin's role in increasing AMPK activation has led to various studies exploring its potential anti‐inflammatory and pro‐autophagy (cellular recycling) effects on various cell lines.^14^, ^19^ Furthermore, metformin has been shown to protect nucleus pulposus cells against apoptosis and senescence via autophagy stimulation and ameliorate disc degeneration in vivo in rodents.^20^ However, there is sparse literature investigating the potential anti‐inflammatory properties of metformin in relation to the intervertebral disc to combat disc degeneration. Therefore, in this study, we hypothesize that metformin may impart its anti‐inflammatory effect on disc cells through the NF‐κB pathway.

### 1.1 Objective

The objective of this study was to investigate the effects of metformin on annulus fibrosus cells under in vitro conditions that mimic biological and mechanical contributions to IDD. A previously published study^4^ showed the effect of inflammatory and mechanical stress on the gene expression of rat AF cells and found that these effects can be attenuated using a NF‐κB inhibitor. Similarly, we hypothesize that Metformin may impart its anti‐inflammatory effect on disc cells through the NF‐κB pathway. Investigation of metformin's anti‐inflammatory properties in disc cells may provide a foundation for further research on metformin's potential therapeutic uses in treating low‐back pain resulting from intervertebral disc degeneration.

## 2 METHODS

Disc cells were subject to metformin under conditions of inflammation, traumatic mechanical stretch, and combined inflammatory and mechanical stimuli to identify the pathways in which metformin interacts with cellular processes, with a particular focus on changes in the inflammatory response and extracellular matrix ‐associated gene expression of AF cells.

### 2.1 Annulus fibrosus cell isolation and culture

Four Fischer 344 rats (mean age: 9 ± 2.5 months) were obtained under IACUC protocol# 21028662. Following euthanization, rats were incised posteriorly from cervical to sacral spine. Rat spines were extracted along with the tail for cell culture. Annulus fibrosus was collected and plated onto T‐75 flasks with F‐12 media and stored in a hypoxic chamber (5% CO_2_, 5% O_2_, 37.5°C) to simulate the intervertebral disc environment. Once cells had grown to ~85% confluence, they were passaged with trypsin and plated onto six‐well FlexCell (FlexCell International Corp., Hillsborough, NC) plates of cell culture slides for subsequent treatment.

### 2.2 Treatment groups

**FIGURE 1:**
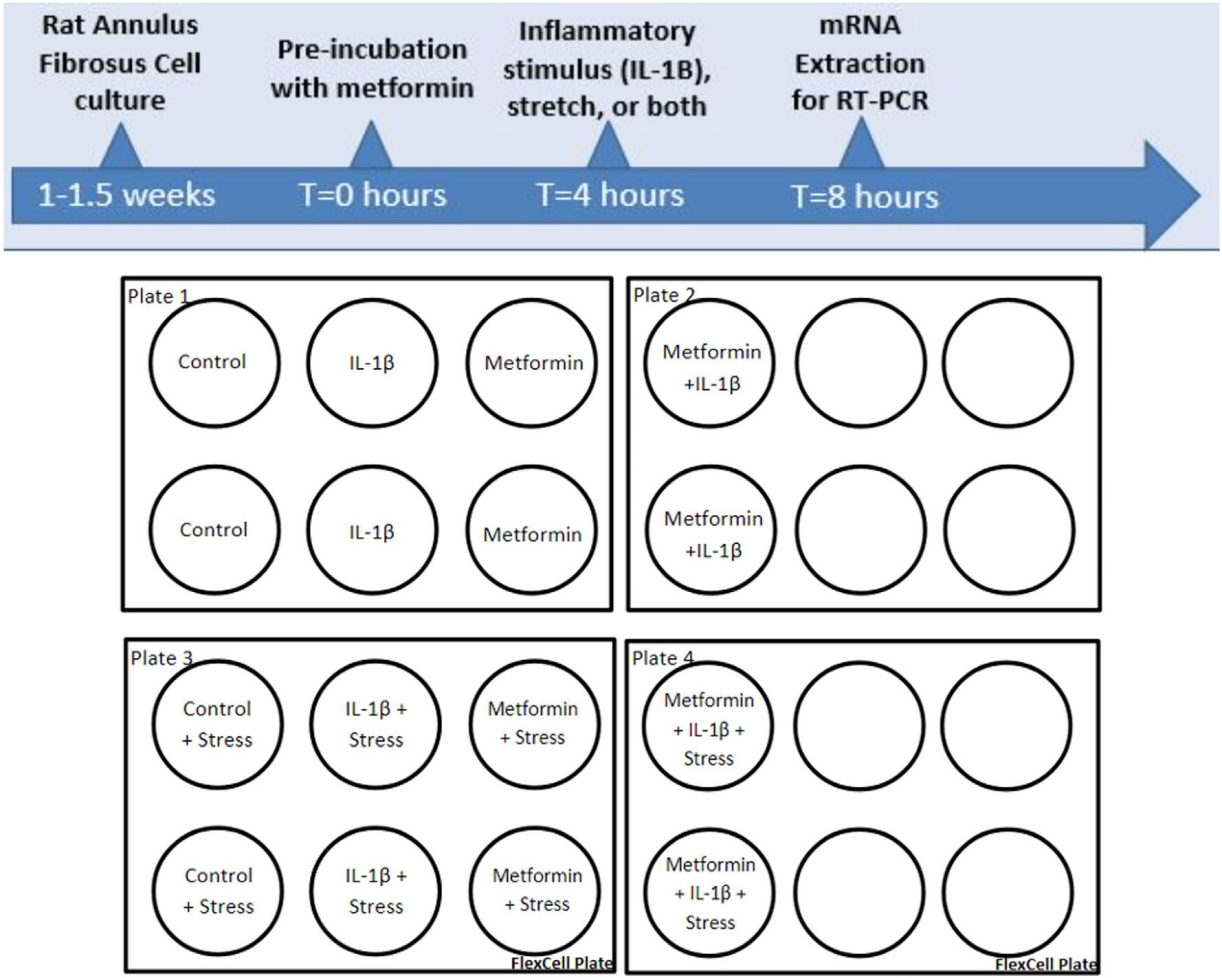
Experimental design depicting metformin pre‐treatment of rat annulus fibrosis (AF) cells prior to inflammatory and mechanical stress. This protocol is followed by mRNA extraction for reverse transcriptase polymerase chain reaction (RT‐PCR)

AF cells are plated onto FlexCell plates or cell culture chamber slides and split into four treatment groups: control, metformin, IL‐1β, and metformin + IL‐1β (Figure 1). Cultures were pretreated with either 100 mM metformin HCl (Sigma‐Aldrich) or F‐12 (1% FBS/1% PS) medium for 4 h in a hypoxic incubator (5% CO_2_, 5% O_2_, 37.5°C). Then, each of the metformin and medium pretreated cells were further treated with 1 ng/ml of IL‐1β (R&D Systems) for 30 min, 4 h or 24 h, respectively. Metformin was added as a pretreatment to allow cells to incorporate metformin sufficiently into their metabolism before other treatments which is a typical study design for studying inhibitors. In addition, pretreatment with metformin is consistent with the typically chronic, not episodic, clinical use of metformin.

### 2.3 Stretch treatment

Following IL‐1β treatment for 4 h, plates with stretch conditions were placed on a FlexCell baseplate (FX‐4000; Flexcell International Corp.) with airtight gaskets and subjected to 24 h of mechanical stretching at 0.5 Hz and 18% strain in the incubator. At *t* = 28 h, all plates were removed from the incubator and treatment media was collected.

### 2.4 RNA isolation and quantitative real‐time polymerase chain reaction

After treatment, cells were lysed with 10:1 solution of RLT Plus:BME. Cell lysate and treatment media were collected and stored at −80°C prior to RNA isolation. The QIAGEN RNeasy Plus Mini Kit (QIAGEN, catalog no. 74134) was used to isolate RNA from cell lysate for all groups. Cell lysates were treated with a DNAse step to eliminate genomic DNA. A nano‐spectrophotometer was used to assess RNA concentration and quality. Quantitative real‐time polymerase chain reaction (qRT‐PCR) was used to measure gene expression with custom‐designed rat primers for MMP‐13, COX‐2, iNOS, Col1, and AGC. PCR data were analyzed using the 2^−ΔΔCt^ method for calculating relative gene expression.^21^ Glyceraldehyde phosphate dehydrogenase (GAPDH) is a constitutively expressed gene shown to be an appropriate housekeeping gene for disc cell mechanobiology testing^22^ and was used as such in all assays. Relative gene expression as a fold‐change and as a percent‐change between groups were compared based on treatment of interest. To explore the effect of induced inflammation under nonmechanical and mechanical conditions, the IL‐1β group was compared to control and IL‐1β/Stretch was compared to Stretch alone, respectively (i.e., IL vs. C and IL/S vs. S). The effect of mechanical stimulation was investigated by comparing stretch conditions with their analog in the nonstretch group (i.e., IL/S vs. IL). Similarly, the effect of metformin was explored by comparing metformin conditions to their analogs in the nonmetformin groups (i.e., M + IL/S vs. IL/S).

### 2.5 PGE_2_ enzyme‐linked immunosorbent assay

PGE_2_ enzyme‐linked immunosorbent assay (ELISA) kit was obtained (KGE004B, R&D Systems) for investigating the presence of PGE_2_ among all treatment groups. Conditioned media from all trials were diluted 3‐fold with calibrator diluent for use. A competitive horseradish‐peroxide (HRP) enzyme probe reporter was used for its high turnover rate and specific enzyme activity. All absorbance values were read using a spectrophotometer (PerkinElmer, Waltham, MA) set to 450 nm for the main reading, and 570 nm for background noise detection.

### 2.6 NF‐κB immunofluorescence and quantification

The cells were stained with rabbit anti‐p65 subunit of NF‐κB primary antibody (1:200 dilution, Cell Signal) and then treated Cy3 Goat anti‐Rb IgG (1:500 dilution, Jackson ImmunoResearch) and stained with the nuclear DAPI stain (ThermoFisher) and cover slipped before being imaged at ×40. These images were analyzed through a quantification software to quantify cytoplasmic and nuclear NF‐κB levels. The ratio of nuclear to total NF‐κB signal between the IL‐1β treatment group and IL‐1β + metformin treatment groups was assessed at each time point since activation of NF‐κB pathway is indicated by p65 nuclear translocation from the cytoplasm.

### 2.7 Statistical analysis

A thorough normality assessment of the raw delta Ct values was completed using the Shapiro–Wilk test for normality. This test was applied to every gene and group to test whether the true difference in gene expression is a departure from the normal distribution. With type I error set at 0.05, all groups were found to follow a normal distribution, suggesting that parametric testing is appropriate for this dataset. Two‐way ANOVA in the stretch and IL‐1β was used to compare treatment groups for each gene with Type I error set at α =0.05. Main column effects were calculated with Dunnett's multiple comparisons test. Statistical analysis of PGE_2_ concentrations among groups was conducted with one‐way ANOVA, with Type I error α =0.05. Inter‐group averages were compared using Tukey's multiple comparisons test. Statistical comparison of NF‐κB quantification was done using a *t*‐test. All statistical analyses were performed in the statistical software Prism 8.0 (GraphPad Software, San Diego, CA).

## 3 RESULTS

### 3.1 Gene expression: Non‐inflammatory conditions

**FIGURE 2:**
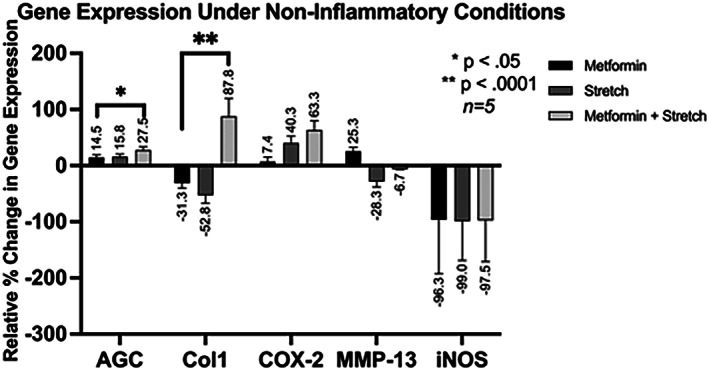
Relative percent change in gene expression under non‐inflammatory conditions with respect to the control group. For example, in the Stretch group, gene expression of AGC was 15.8% greater than in the control group

Figure 2 presents the gene expression of AGC, COX‐2, MMP‐13, Col1, and iNOS under non‐inflammatory conditions.

Metformin in the presence of stretch significantly increased matrix component AGC gene expression (*p* <0.05) and Col1 gene expression (*p* <0.0001) compared to metformin alone (M). Metformin and stretch stimulus upregulated pro‐inflammatory COX‐2 expression by a larger magnitude than either metformin or stretch condition alone. While metformin alone (M) increased pro‐inflammatory MMP‐13 expression by 25.3%, the contribution of stretch stimulus (M + S) decreased expression to −6.7% relative to control, although this difference was not statistically significant. These gene expression data under noninflammatory conditions suggest that metformin's protective effects may be enhanced by mechanical stretch.

### 3.2 Gene Expression: Inflammatory conditions

**FIGURE 3:**
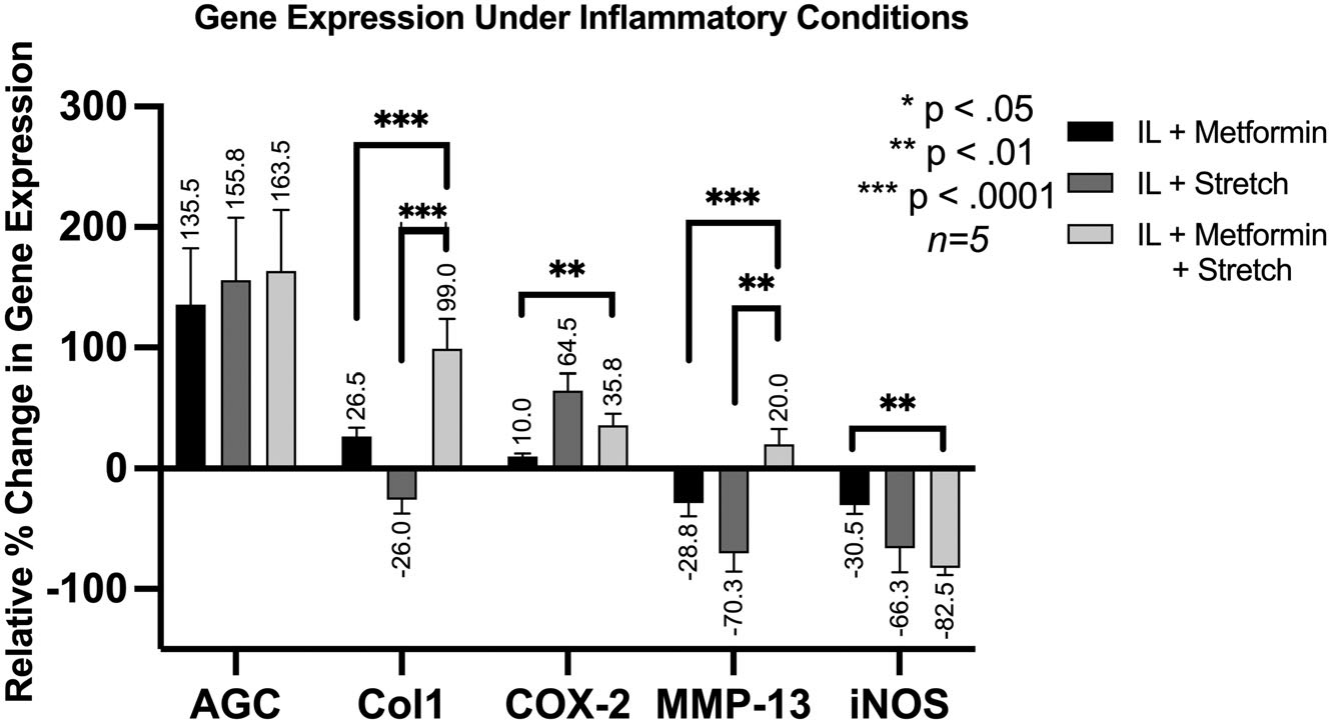
Relative percent change in gene expression under inflammatory (IL‐1β) conditions with respect to the control group. For example, in the IL + metformin group, gene expression of AGC was 135.5% greater than in the control group

Figure 3 presents the gene expression of AGC, COX‐2, MMP‐13, Col1, and iNOS under inflammatory conditions.

The addition of metformin to conditions of combined inflammatory and mechanical stretch (M + IL/S) increased matrix component Col1 gene expression compared to IL/S conditions alone (*p* <0.001). In addition, metformin under conditions of combined inflammatory and mechanical stretch (M + IL/S) increased pro‐inflammatory MMP‐13 gene expression compared to IL/S conditions alone (*p* <0.01). In addition, metformin in the presence of IL + S resulted in increased Col1 (*p* <0.0001), increased COX‐2 (*p* <0.01), increased MMP‐13 (*p* <0.0001), and decreased iNOS (*p* <0.01) compared to metformin in the presence of IL alone. These gene expression data suggest that metformin's protective effects may be enhanced by mechanical stretch even under inflammatory conditions.

### 3.3 Prostaglandin E2 detection and quantification

We next studied whether metformin impacts inflammatory mediator expression. PGE_2_ was measured as a downstream inflammatory mediator modulated by COX‐2 expression.

**FIGURE 4:**
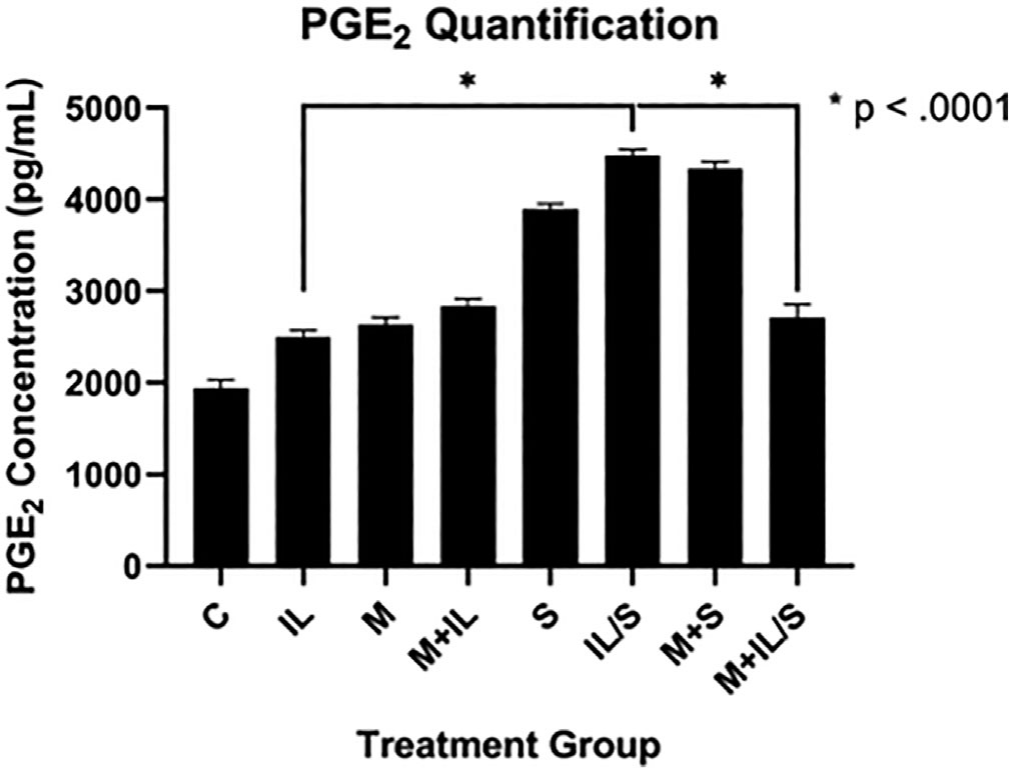
Prostaglandin E2 (PGE2) concentration of all groups in pg/ml

PGE_2_ concentration was lowest in the control group (1941 pg/ml) and peaked during IL/S treatment group (4481 pg/ml). Combined stress treatments (IL/S) significantly increased PGE_2_ expression (*p* <0.0001) compared to inflammatory stress alone. The presence of metformin under combined stress treatments (M + IL/S); however, significantly decreased PGE_2_ expression (*p* <0.0001) to 2714 pg/ml from the 4481 pg/ml observed in the IL/S group (Figure 4). However, metformin did not significantly reduce PGE_2_ expression under IL conditions alone. This trend was also not observed under noninflammatory conditions, where the addition of metformin to mechanical stretch (M + S) increased PGE_2_ expression modestly from 3898 to 4341 pg/ml. These data suggest that metformin produces anti‐inflammatory effects under combined inflammatory and mechanical stress at the inflammatory mediator level in the disc annulus fibrosus cells.

### 3.4 NF‐κB immunofluorescence

**FIGURE 5:**
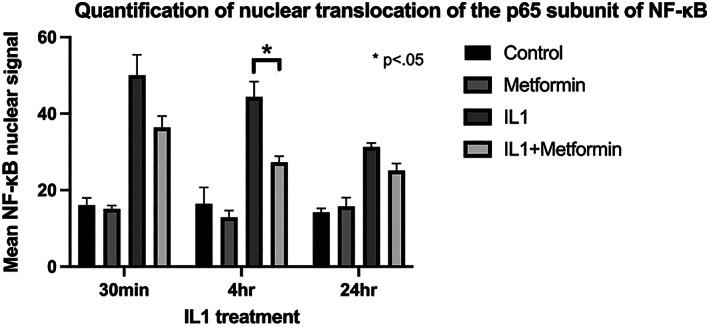
Quantification of nuclear translocation of the p65 subunit of NF‐κB. Quantification is expressed as a ratio of nuclear to total NF‐κB signal

We investigated NF‐κB as a potential pathway metformin uses to exert its anti‐inflammatory effects. The impact of combined stretch and inflammatory stress on AF cells has been previously studied and demonstrated that the effects of combined stressors can be attenuated using an NF‐κB inhibitor.^4^ Similarly, we hypothesize that metformin produces its anti‐inflammatory effect through the NF‐κB pathway. In Figure 5, metformin treatment of cultured rat annulus fibrosus cells significantly reduced the nuclear translocation of NF‐κB (*p* <0.05) after 4 h of IL1‐beta treatment from 43.1% in case of IL‐1β treatment down to 26.2% in the case of metformin + IL‐1β treatment. Metformin treatment also reduced nuclear translocation of NF‐κB (n.s.) after 30 min and 24 h of IL1‐beta treatment from 52.1% to 36.76% and from 31.4% to 25.7%, respectively, but these results were not statistically significant. These data suggest that NF‐κB mediates the anti‐inflammatory effects of metformin in AF cells.

## 4 DISCUSSION

Our data suggest that the anti‐inflammatory effect of metformin in disc cells may be mediated through the NF‐κB pathway. In addition, metformin's effects appear to be enhanced under mechanical stretch conditions.

The difference in gene expression profiles between noninflammatory and inflammatory conditions highlights key differences that may provide insight into metformin's mechanism of action. Metformin in the combined presence of mechanical stretch and IL‐1β (M + IL/S) suppressed COX‐2 (Figure 3), but substantially increased COX‐2 mRNA transcripts in the noninflammatory (M + S) group (Figure 2). Similarly, PGE_2_ expression is reduced under M + IL/S conditions but increased under M + S conditions. Similar to our study, the Neidlinger–Wilke group found that combined inflammatory and mechanical stress synergistically increases PGE2 and COX2 expression.^23^ Our study finds that metformin significantly reduces PGE2 and COX2 expression in the presence of this synergistic stress. Since COX‐2 and PGE_2_ expression are decreased under M + IL/S condition and increased under M + S condition, we demonstrated that metformin may not be acting to enzymatically inhibit COX‐2, unlike traditional NSAIDs, but instead it may be acting through the NF‐κB pathway to modulate COX‐2 expression.

As previously described, NF‐κB is a prominent mediator in mechanical and inflammatory stress signaling pathways. Mechanical stimulation upregulates nuclear translocation of NF‐κB, resulting in transcription of a range of inflammatory cytokines, including the production of IL‐1β.^4^ When metformin was added to IL‐1β treated rat annulus fibrosus cells, it downregulated the nuclear translocation of NF‐κB (Figure 5), suggesting metformin does indeed produce its effects in part through the NF‐κB pathway.

Although the inflammatory response seems synergistic and positively reinforced between stretch and IL‐1β as previously described by Miyamoto,^6^ the stretch condition seems to be a greater contributor to inflammation compared to IL‐1β alone. Based on our findings, metformin blunts the stretch‐mediated inflammatory response at least in part through NF‐κB inhibition. Metformin may also play a mediatory role in PGE_2_‐related inflammation in the disc (Figure 5).

The homeostasis of disc cells is closely governed by the balance of anabolic and catabolic factors. Metformin increases Col1 expression under inflammatory conditions (M + IL). Furthermore, in both inflammatory and noninflammatory conditions, metformin increases Col1 production even more through the combined effects of metformin and stretch (M + IL/S and M + S groups) (Figures 2 and 3). These data suggest that metformin's mechanism of action on anabolic gene expression is at least partially dependent on mechanical signaling pathways. The upregulation of Col1 in combined stress and metformin treatments implies improved ECM production, which is crucial for balancing catabolic factors that simultaneously degrade the disc. Another study also determined that annular delamination strength, measured in N/mm, was lowest in the presence of combined chemical and mechanical stress.^23^ Metformin's enhanced effects under mechanical stretch conditions shows potential in slowing the degenerative process by maintaining delamination strength in intervertebral disc cells. Further studies examining Metformin's role in annular delamination strength are warranted.

### 4.1 Implications

To our knowledge, metformin has been sparsely explored as a potential therapeutic agent for disc degeneration. Our findings provide evidence to suggest that metformin may be a viable anti‐inflammatory alternative to NSAIDs. From gene expression analysis and downstream PGE_2_ quantification, it is possible that metformin acts at the gene transcription level through the NF‐κB pathway to curb inflammation and simultaneously improve matrix production. In this way, metformin's mechanism of action is different compared to the currently prescribed COX‐1 and ‐2 inhibitors, which act as enzymatic inhibitors. Metformin's anti‐inflammatory effects may prove useful not only for treating disc degeneration, but also as a system‐wide anti‐inflammatory agent with potential for ameliorating a plethora of inflammation‐related conditions.

## CONFLICT OF INTEREST

None declared.

## AUTHOR CONTRIBUTIONS

Each author has read and approved the final submitted manuscript. Rahul Ramanathan contributed to conception and design, data acquisition, data interpretation, manuscript drafting, manuscript editing, and statistical analysis. Ayesha Firdous contributed to data acquisition, data interpretation, manuscript drafting, manuscript editing, and statistical analysis. Qing Dong contributed to data acquisition and data interpretation. Dong Wang contributed to data acquisition, data interpretation, and supervision. Joon Lee contributed to obtaining funding, supervision, and manuscript editing. Nam Vo contributed to conception and design, supervision, and obtaining funding. Gwendolyn Sowa contributed to conception and design, obtaining funding, supervision, data interpretation, and critical revisions.
